# The Effects of Vitamin D Supplement on Prevention of Recurrence of Preeclampsia in Pregnant Women with a History of Preeclampsia

**DOI:** 10.1155/2017/8249264

**Published:** 2017-08-17

**Authors:** Sanam Behjat Sasan, Farnaz Zandvakili, Nasrin Soufizadeh, Elaheh Baybordi

**Affiliations:** ^1^Department of Gynecology, School of Medicine, Kurdistan University of Medical Science, Sanandaj, Iran; ^2^East Azerbaijan ACECR Medical Center, Tabriz, Iran

## Abstract

**Introduction:**

Preeclampsia is a pregnancy-specific syndrome. One of the hypotheses concerning the etiology of preeclampsia is vitamin D deficiency during pregnancy.

**Method and Materials:**

The present study is a randomized controlled clinical trial which aims to determine the effect of vitamin D supplement on reducing the probability of recurrent preeclampsia. 72 patients were placed in control group while 70 patients were randomized to the intervention group. The intervention group received a 50000 IU pearl vitamin D3 once every two weeks. The control group was administered placebo. Vitamin D or placebo was given until the 36th week of pregnancy.

**Results:**

The patients in intervention group have significantly lower (*P* value = 0.036) probability of preeclampsia than patients in the control group. The risk of preeclampsia for the control group was 1.94 times higher than that for the intervention group (95% CI 1.02, 3.71).

**Conclusion:**

The intended intervention (i.e., prescription of vitamin D) has a protective effect against recurrent preeclampsia. Vitamin D supplementation therapy in pregnancy could help in reducing the incidence of gestational hypertension/preeclampsia.

**Registration:**

This study has been registered in Iranian Registry of Clinical Trials (IRCT) site with ID number IRCT2017010131695N1.

## 1. Introduction

Preeclampsia is pregnancy-specific syndrome, characterized by high blood pressure induced and proteinuria after 20 weeks of gestation. It complicates 2–8% of all pregnancies and accounts for 25% of all maternal deaths and perinatal morbidity and mortality. Although preeclampsia is something more than simple gestational hypertension with proteinuria, development of proteinuria is still one significant and objective diagnostic measure of this disorder. Proteinuria is defined as excretion of more than 300 mg of protein in 24-hour urine collection, protein-creatinine ratio of 0.3 or higher in random urine samples, or consistent amount of protein (i.e., 30 mg per deciliter) in randomly taken samples of urine (i.e., +1 result on dipstick) [1].

Disorders of calcium metabolism, including hypocalciuria and low vitamin D level, have been consistently described, during in the course of pregnancy of women who later developed preeclampsia [2–4].

Factors contributing to preeclampsia are diabetes, chronic hypertension before pregnancy, chronic kidney diseases, nulliparity, twin or multiple pregnancy, family history of preeclampsia or eclampsia, obesity, immune disorders and a personal history of preeclampsia, or eclampsia. The occurrence of preeclampsia in one pregnancy does not necessarily predict the occurrence of preeclampsia in subsequent pregnancies. However, its initial development is associated with a higher probability of it occurring in subsequent pregnancies.

Vitamin D is especially important during pregnancy as low maternal vitamin D stores may contribute to problems such as low birth weight and small for gestational age infants, as well an increased risk of maternal comorbidities [5].

Vitamin D deficiency is worldwide epidemic, with a prevalence that ranges from 18% to 84% depending on the country of residence, ethnicity, and local clothing customs and dietary intake [6, 7]. Clinical studies establishing an association between vitamin D levels and adverse pregnancy outcomes such as preeclampsia, gestational diabetes, and low birth weight, preterm labor, and caesarean delivery have conflicting results [8].

Previous studies have confirmed that low level of vitamin D disrupts the balance between Th1 and Th2 and contributes to overexpression of Th1 cytokines. The latter event affects immunological tolerance of embryo implantation. The studies suggest that deficiency of vitamin D could be associated with higher expression of Th1 which is observed in cases of preeclampsia [9].

There are different hypotheses concerning the etiology of preeclampsia, one of which is vitamin D deficiency in pregnancy. In the present study, vitamin D supplement was administered to pregnant women with history of preeclampsia in previous pregnancies. Considering the fact that one of the possible etiologies of preeclampsia is the increased requirement of vitamin D during pregnancy, that increased need is satisfied by prescribing vitamin D supplement and will allow us to examine its role in preventing preeclampsia.

## 2. Method and Materials

The present study is a randomized controlled clinical trial which aims to determine the effect of vitamin D supplement on reducing the probability of recurrent preeclampsia in pregnant women with history of preeclampsia.

The study population included women who were referred to the obstetrical clinic in Besat Hospital of Sanandaj City who were receiving prenatal care and had a history of preeclampsia in previous pregnancies. In the case of willingness to participate in the present study, they were given agreement forms to fill in and their serum levels of vitamin D3 were analyzed. If a participant's level of 25-hydroxy vitamin D was equal or higher than 25 ng/ml (i.e., normal range), she was considered a candidate for the study (inclusion criteria). Risk of chronic hypertension before pregnancy, concurrent renal, pulmonary and cardiac diseases, immunologic diseases such as lupus, lack of confidence in patient's cooperation to complete study, and immigration or leaving location of study were regarded as exclusion criteria.

After satisfaction of inclusion and exclusion criteria, simple randomization and blinding were done concurrently. In this regard, 140 pockets of drug and placebo were randomly (by using table of random numbers) offered and neither physician nor patients knew about administration of drug or placebo.

After inclusion in the present study, blood samples of all patients were taken to analyze level of vitamin D. After 12 hours of fasting, level of vitamin D was determined through Liebermann–Burchard method. After obtaining consent for participation blood was sent to the laboratory for vitamin D analysis. Once the patient was determined to be eligible for the study the study drug was started, the intervention group received a 50000 IU pearl vitamin D3 once every two weeks. The control group was administered placebo. Both groups received a study drug (vitamin D or placebo) until the 36th week of pregnancy. The drug and placebo were both purchased from Zahravi Pharmaceutical Company.

Identification of patients with preeclampsia was done through clinical examination and review of laboratory results (e.g., blood pressure of 140/90 mm Hg or higher in sitting position) and proteinuria of higher than +1. Blood pressure was measured every two weeks while receiving the study drug. If blood pressure was equal to or higher than 140/90 mm Hg in sitting position, urine test for proteinuria was requested. In the case of observing normal blood pressure, the patient was reexamined two weeks later.

Through SPSS software (version 16), independent *t*-test of normal quantitative variables was conducted for both independent groups. In addition, chi-square test was conducted for comparison of nominal variables of the two groups. Controlling other factors, logistic regression was done to compare development of preeclampsia in both groups.

## 3. Results

Total number of study participants was 142 individuals who had satisfied inclusion criteria. The participants were randomly placed into two groups (i.e., intervention group and control group). Consequently, 72 patients were placed in control group while 70 patients were classified into intervention group. The baseline characteristics of both groups are shown in Table 1.

In intervention group, all patients had singleton pregnancy while two cases in the control group (2.8 percent) had twin pregnancy. Two cases (2.9 percent) from intervention group and 4 cases (5.6 percent) in the control group had married for second time and the rest of participants had married once.

In regard to residence location, 20 individuals (28.6 percent) in the intervention group were living in villages and the remaining 50 individuals (71.4 percent) of the intervention group were urban residents. In the control group, one could state that 23 individuals (31.9 percent) were rural residents and the remaining 49 individuals (68.1 percent) were urban residents. Positive history of diabetes was found in 4 patients (5.6 percent) in the intervention group while there was no such a history in the control group. No history of cardiac diseases, gestational hypertension, high blood pressure, thyroid disease, immunological disorders, lung diseases, and renal disorders was found for patients in either group. None of the participants had a history of consuming vitamin D supplement. Family history of preeclampsia was negative for all patients. In regard to fetal health, 48 patients (72.7 percent) in the intervention group and 62 patients (87.5 percent) in the control group were screened.

None of the subjects reported side effects. There were cooperation and adherence of all study participants with taking the study drug.

The comparison of termination of pregnancy by normal vaginal delivery or caesarean section or abortion is shown in Table 2.

The final outcome of the present study was recurrence of preeclampsia in the intervention group and control group. The patients in the intervention group have significantly lower (*P* value = 0.036) probability of preeclampsia than patients in the control group. The relevant results are shown in Table 3.

One could state that the risk of preeclampsia for the control group was 1.94 times higher than for intervention group (95% CI 1.02, 3.71).

## 4. Discussion

The results of the present study suggest that prescription of vitamin D supplement in the first trimester of pregnancy contributes to preventing recurrence of preeclampsia (*P* value = 0.036). In regard to effects of vitamin D on preeclampsia, evidence suggests that vitamin D metabolism is associated with preeclampsia.

There are many biologically acceptable mechanisms by which the maternal vitamin D status can impact the risk of preeclampsia.

Preeclampsia is a pregnancy complication with serious consequences. The disease is diagnosed by the presence of gestational hypertension and proteinuria. Preeclampsia is proposed to occur in 2 stages [10]. In stage 1 placental perfusion is reduced. This could happen following an abnormal implantation. The poor blood flow through the placenta is proposed to produce substances that in a favorable maternal environment initiate the ensuing multisystem abnormalities (stage 2). The endothelial dysfunction is part of generalized intravascular inflammatory reaction involving leukocytes and the clotting and complement systems. It seems that poor placental blood flow is not the main cause of preeclampsia but it is a powerful predisposing factor [11]. The active form of vitamin D, 1,25-dihydroxyvitamin D_3_, has been demonstrated to adjust the transcription and function of genes associated with normal implantation, placental invasion, and angiogenesis [12]. The immunomodulatory properties of 1,25-dihydroxyvitamin D are relevant. Abnormal implantation is proposed to be mediated at least in part by an abnormal immune response between pregnant mother and infant [13]. Maternal vitamin D deficiency may increase the inflammatory reaction [14]. Vitamin D deficiency may also increase the risk of hypertension [15].

Finally, renal vascular endothelial growth factor (VEGF) seems to be associated with proteinuria. 1,25-Dihydroxyvitamin D_3_ could regulate angiogenic processes through effects on VEGF gene transcription [16].

Vitamin D deficiency, as measured by 25-hydroxyvitamin D [25(OH) D] serum levels are common in pregnant women. A positive correlation between vitamin D level and adverse pregnancy outcomes such as preeclampsia, preterm birth, and gestational diabetes mellitus was shown in several meta-analyses of observational studies [17].

Many studies have shown that the risk of preeclampsia is increased when vitamin D serum level is low [18]. Normal level of 1,25 dihydroxyvitamin D may prevent preeclampsia by its effect on immune modulation and vascular function. The National Institutes of Health has funded many clinical trials that aim at determining the effect of vitamin D supplementation during pregnancy and prevention of adverse pregnancy outcomes [19].

A significant association between vitamin D deficiency and preeclampsia has been previously reported (odds ratio, 4.2; 95% confidence interval, 1.4–12.8; *P* value, 0.04) [20].

In one study the prevalence of vitamin D deficiency was very high with more than 3 quarters (78%) of all participants having a serum 25(OH) D level < 30 ng/ml. The mean serum 25(OH) D level was 24.86 ng/ml in normal pregnancies (*N* = 76), 23.96 ng/ml in preeclamptic women (*N* = 33), and 21.56 ng/ml in eclamptic women (*N* = 79). Compared to those women who had a serum 25(OH) D level of ≥30 ng/ml, the odds ratios (95% CI) of developing preeclampsia and eclampsia in pregnant women with vitamin D deficiency were 3.9 (95% CI = 1.18–12.87) and 5.14 (95% CI = 1.98–13.37), respectively (adjusting for age, BMI, and duration of pregnancy) [21].

A recent meta-analysis has demonstrated a correlation between vitamin D and preeclampsia in various study types. They show that vitamin D could act as a preventive factor for preeclampsia [22].

Two clinical trials suggested that vitamin D has a potential role in the prevention of preeclampsia, but neither of them is treated with vitamin D only. In one, supplementation with a multivitamins and minerals supplement and halibut liver oil (containing 900 IU/d vitamin D) provided from 20-week gestation reduce the odds of preeclampsia by 32% (95% CI, 11–47%) [23]. In the other randomized trial 400 women treated with vitamin D (1200 IU/d) and calcium (375 mg/d) supplements or placebo at 20–24-week gestation experienced a significant reduction in blood pressure (*P* < 0.001) and a nonsignificant reduction in the incidence of preeclampsia in the treated group compared with the placebo group (6 versus 9%) was seen [24].

UV-B rays (290 to 310 nm) received by a person on exposed body surfaces induce vitamin D synthesis by the skin [25]. An extremely significant association between vitamin D level and duration of sun exposure has been reported.

Pregnant and nonpregnant women receive much less amount of sunlight especially in Islamic countries due to traditional norms and customs, as well as governmental rules.

A randomized controlled trial compared the daily administration of 400, 2000, or 4000 IU of vitamin D in pregnant women starting at 12 to 16 weeks of pregnancy until childbirth.

Prescription of vitamin D supplement of 4000 IU daily is more efficient in maintaining normal plasma level of 25(OH) vitamin D (>32 ng/ml) without any toxicity [26].

The risk of preeclampsia recurrence is increased in women with a history of preeclampsia. Maternal and neonatal complications are more common in cases of recurrent preeclampsia when compared to the initial episode [27].

Vitamin D is a promising candidate for preeclampsia prevention, and there is an urgent need for well-controlled randomized trials to test its effectiveness and safety.

## 5. Conclusion

Vitamin D deficiency is highly prevalent in all parts of the world. Pregnant women and neonates are highly vulnerable to vitamin D deficiency.

Vitamin D supplementation therapy in pregnancy could help in reducing the incidence of gestational hypertension/preeclampsia.

## Figures and Tables

**Table 1:** Baseline characteristics of participants per group.

Baseline characteristic variables	Intervention group (*n* = 70)	Control group (*n* = 72)	Meaningful level (*P* value)
Age (mean ± SD)	32.04 ± 5.901	29.77 ± 5.21	0.017
Number of previous pregnancies (mean ± SD)	3.04 ± 1.13	2.92 ± .900	0.463
Weeks of pregnancy (mean ± SD)	14.39 ± 3.12	14.39 ± 2.69	0.997
Systolic blood pressure (mm Hg; mean ± SD)	115.87 ± 14.52	114.51 ± 7.27	0.028
Diastolic blood pressure (mm Hg; mean ± SD)	74.28 ± 4.95	74.31 ± 6.40	0.975
Uterine ↓ cm (mean ± SD)	14.58 ± 3.50	14.28 ± 3.26	0.597
24 h proteinuria (mg/cc; mean ± SD)	132.22/1844.91 ± 61.447	154.94/1958.53 ± 53.376	0.023
BMI < 18.5 kg/m^2^ (*n* (%))	1 (1.4%)	1 (1.4%)	0.267
BMI, 18.5–24.9 kg/m^2^ (*n* (%))	23 (32.9%)	14 (19.4%)	0.267
BMI, 25.0–29.9 kg/m^2^ (*n* (%))	32 (45.7%)	35 (49.3%)	0.267
BMI ≥ 30.0 kg/m^2^ (*n* (%))	13 (18.6%)	21 (29.6%)	0.267

↓: uterine height is measured as the distance between the midpoint of the pubic bone and highest peak of the uterus in cm, while the pregnant woman after voiding was lying in the supine position.

**Table 2:** Comparison of pregnancy types and end of pregnancy for intervention and control groups.

Group	Termination of pregnancy			*P* value
	NVD	C/S	Abortion	
Control group	43 (59.7%)	27 (37.5%)	2 (2.8%)	0.88
Intervention group	33 (47.1%)	37 (52.9%)	0 (0%)	
Total	76 (53.5%)	64 (45.1%)	2 (1.4%)	

*P* value based on *Fisher's exact tests*; *P* value < 0.05 is statistically significant.

**Table 3:** Comparison of preeclampsia incidence between intervention group and control group.

Group	Nonpreeclampsia	Preeclampsia	*P* value
Control group	50 (69.4%)	22 (30.6%)	0.036
Intervention group	59 (84.3%)	11 (15.7%)	
Total	109 (76.8%)	33 (23.2%)	

*P* value based on Chi-square tests; *P* value < 0.05 is statistically significant.

## Additional Points

*Limitations*. During the present study, some participating patients were discouraged of participation in the study by nonmedical people and even some of clinical colleagues on alleged safety issues. However, a second briefing meeting was held to address the concerns and encourage the patients to continue their participation. Because gynecologist might recommend patients to stop taking drugs, a briefing meeting was held with some major gynecologists to address some misinterpretations in regard to prescription of the drug.
